# Prolonged cardiovascular pharmacological support and fluid management after cardiac surgery

**DOI:** 10.1371/journal.pone.0285526

**Published:** 2023-05-11

**Authors:** Loay Kontar, William Beaubien-Souligny, Etienne J. Couture, Matthias Jacquet-Lagrèze, Yoan Lamarche, Sylvie Levesque, Denis Babin, André Y. Denault

**Affiliations:** 1 Department of Anesthesiology, Montreal Heart Institute, Université de Montréal, Montreal, Quebec, Canada; 2 Division of Intensive Care Unit, Centre Hospitalier de l’Université de Montréal, Montreal, Quebec, Canada; 3 Medical Intensive Care Unit, Centre Hospitalier Universitaire d’Amiens, Amiens, France; 4 Division of Nephrology, Centre Hospitalier de l’Université de Montréal, Montreal, Quebec, Canada; 5 Department of Anesthesiology and Intensive Care Medicine, Institut Universitaire de Cardiologie et de Pneumologie de Québec, Université Laval, Quebec, Quebec, Canada; 6 Department of Surgery, Montreal Heart Institute, Université de Montréal, Montreal, Quebec, Canada; 7 Montreal Health Innovations Coordinating Centre, Montreal Heart Institute, Montreal, Quebec, Canada; BSMMU: Bangabandhu Sheikh Mujib Medical University, BANGLADESH

## Abstract

**Objective:**

To identify potentially modifiable risk factors related to prolonged cardiovascular pharmacological support after weaning from cardiopulmonary bypass (CPB).

**Methods:**

This is a secondary analysis of two prospective cohort study in a specialized cardiac surgery institution in adult patients undergoing cardiac surgery with the use of CPB between August 2016 and July 2017. Prolonged cardiovascular pharmacological support was defined by the need for at least one vasopressor or one inotropic agent 24 hours after separation from CPB. Risk factors were identified among baseline characteristics and peri-operative events through multivariable logistic regression.

**Results:**

A total of 247 patients were included and 98 (39.7%) developed prolonged pharmacological support. In multivariable analysis, left ventricular ejection fraction ≤ 30% (OR 9.52, 95% confidence interval (CI) 1.14; 79.25), elevated systolic pulmonary artery pressure (sPAP) > 30 and ≤ 55 mmHg (moderate) (OR 2.52, CI 1.15; 5.52) and sPAP > 55 mmHg (severe) (OR 8.12, CI 2.54; 26.03), as well as cumulative fluid balance in the first 24 hours after surgery (OR 1.76, CI 1.32; 2.33) were independently associated with the development of prolonged pharmacological support.

**Conclusions:**

Prolonged cardiovascular pharmacological support is frequent after cardiac surgery on CPB. Severe LV systolic dysfunction, preoperative pulmonary hypertension and postoperative fluid overload are risk factors. Further studies are required to explore if those risk factors could be modified or not.

## Introduction

Hemodynamic instability is a frequent complication after cardiopulmonary bypass (CPB) separation and can lead to significant morbidity and mortality that worsen postoperative clinical outcomes [1, 2]. Vasoplegia syndrome (VS) [3] and low cardiac output syndrome (LCOS) [4] are the most common causes of prolonged cardiovascular pharmacological support after weaning from CPB. The incidence of VS vary from 5% to 45% of patients [5] while LCOS is around 3.9% to 14.7% [4, 6–8]. This wide variation is due to some extent to the absence of consensual definitions in terms of threshold or duration. Even in the absence of LCOS, VS can lead to prolonged use of vasoconstrictor agents with increased mortality [9, 10].

Only limited evidence is available regarding risk factors of prolonged cardiovascular pharmacological support following cardiac surgery [11]. Several risk factors have been identified such as CPB duration [9, 11], platelet transfusion [12], lower temperature during CPB [13], an elevated interleukin-6 level 4 hours after CPB [11] and reduced left ventricular ejection fraction (LVEF) [11]. A reduced hematocrit, which may be mediated by fluid overload, has not been reported as a risk factor [9].

The identification of potentially modifiable risk factors for prolonged cardiovascular pharmacological support would provide the opportunity to investigate strategies to reduce the incidence of this complication. The primary objective of this study was to explore reversible factors associated with prolonged cardiovascular support after CPB and to determine if they would anticipate this complication when included in a predictive model.

## Methods

Montreal Heart Institute Ethics Committee approved the protocol (No. F11C-11495).

The data of this retrospective study are collected from two observational prospective studies (clinicaltrials.org identifier: NCT02658006 and NCT02831907) conducted between August 2016 and July 2017 for which all patients had given their written consent.

### Study setting and patient selection

Patient data was collected from two observational prospective studies conducted between August 2016 and July 2017 [14, 15], in which repeated echocardiographic evaluation including intraoperative transesophageal echocardiography (TEE) and postoperative bedside transthoracic echocardiography were performed. This study was conducted in accordance with the amended Declaration of Helsinki. The study included adult patients (≥18 years old) who underwent elective cardiac surgery with CPB. Exclusion criteria included heart transplantation and usage of ventricular assist devices before and after CPB, and patients requiring antihypertensive drugs after CPB weaning.

### Data sources

Data including laboratory tests, surgical and anesthetic variables as well as drug dosages were retrieved from the electronic patient record and the electronic preoperative anesthesia record (CompuRecord, Philips, Netherlands).

### Definitions

Prolonged cardiovascular pharmacological support was defined as the need for at least one vasopressor or one inotropic agent from the end of CPB for a duration greater than 24 hours which prevent discharge from the intensive care unit (ICU). Vasopressor and inotropic agents included norepinephrine, vasopressin, epinephrine, dopamine, dobutamine, milrinone and phenylephrine. Other clinical variables included the duration of vasoactive and mechanical ventilation support, ICU and hospital length of stay, and early complications occurring in the ICU, including acute kidney injury (AKI), use of hemodialysis, delirium and death in the first 30 days after ICU admission Definition of variables are provided in the S1 Table.

### Data collection

Data regarding the following preoperative variables were collected: demographics, comorbidities and preoperative medication. Intraoperative variables included CPB duration, aortic cross clamp duration, nature of the surgical procedure, cumulative dosage of vasopressors during the intervention, use of pulmonary vasodilatory agents before and after separation from CPB, intraoperative fluid balance, minimal hematocrit level reached, and categorization of CPB weaning [16]. Source of arterial blood pressure monitoring was noted whether the arterial line for pressure monitoring was place centrally in the femoral artery or peripherally in the radial artery. The use of continuous processed electroencephalographic (pEEG) monitoring to monitor anesthesia level was also noted [17]. Until the first 24 hours after surgery: hourly fluid balance and ICU fluid balance, maximum lactate level, and vasopressor or inotrope treatment, including the dose and duration of therapy. Cumulative fluid balance at the end of postoperative day 1 was also collected. All patients included had baseline and repeated portal Doppler assessments performed for research purposes, as previously reported [14, 15, 18].

### Standard intraoperative management at the institution

The anesthesia was composed of propofol, rocuronium, and fentanyl or sufentanil for induction and a continuous infusion of propofol with either fentanyl or sufentanil with isoflurane or sevoflurane for maintenance. Diuretics and hemofiltration were used in case of fluid overload and venous congestion [18], and inhaled vasodilators before CPB were usually considered (based on the anesthesiologist’s decision) in patients with pulmonary hypertension (PH) [19]. Mechanical ventilation was administered with tidal volumes of 6–8 mL/kg. Continuous pEEG monitoring (Sedline, Masimo, Irvine, CA, USA) was introduced in February 2017 for all patients at the institution. Intraoperative monitoring included a 5-lead electrocardiogram, pulse oximetry, cerebral near-infrared spectroscopy, central venous pressure or pulmonary artery pressure (PAP) catheter according to the anesthesiologist preference, as well as invasive blood pressure monitoring using radial or both radial and femoral artery catheters [20]. Perioperative TEE was performed before and after CPB by cardiac anesthesiologists certified by the National Board of Echocardiography. All TEE images were acquired according to the American Society of Echocardiography and the European Association of Echocardiography recommendations [21]. In addition, the choice of the appropriate therapy was based on the best available evidence using a previously reported vasoactive and CPB weaning protocol [22].

### Statistical analysis

Characteristics of patients were presented according to whether they developed prolonged cardiovascular pharmacological support or not. Continuous variables were reported as mean (standard deviation, SD) when normally distributed or as median (interquartile range, [IQR]) when non-normally distributed. Categorical variables were reported as proportion. Groups were compared with Student t or Mann-Whitney rank test, or Chi-square test, as appropriate. Associations between preoperative, intraoperative and postoperative variables, and the risk of prolonged cardiovascular pharmacological support were assessed using univariate logistic regression analysis. A multivariable logistic regression model was then constructed. Variable selection was performed using a backward stepwise selection approach. In any case, basic assumptions were checked prior to analysis. Internal validation of the multiple logistic model was done using a bootstrapping procedure. Two hundred bootstrap samples were generated. Discrimination (optimism, C-statistics) and calibration of the slope were reported.

As exploratory analyses, for variables related to fluid balance including CFB at day 1 and intraoperative fluid balance, the linearity of the logit assumption was tested using the Box-Tidwell test [23]. If a significant deviation from the assumption was found, the relationship between the continuous variable and the risk of prolonged vasopressor dependence was graphically represented by using by a locally estimated scatterplot smoothing (LOESS) regression line using the ggplot2 R package. All *P* values < 0.05 were considered statistically significant. Analyses were performed with both, SAS release 9.4 (SAS Institute Inc., Cary, NC, USA) and R (R Core Team, Vienna, Austria) programs.

## Results

**Fig 1:**
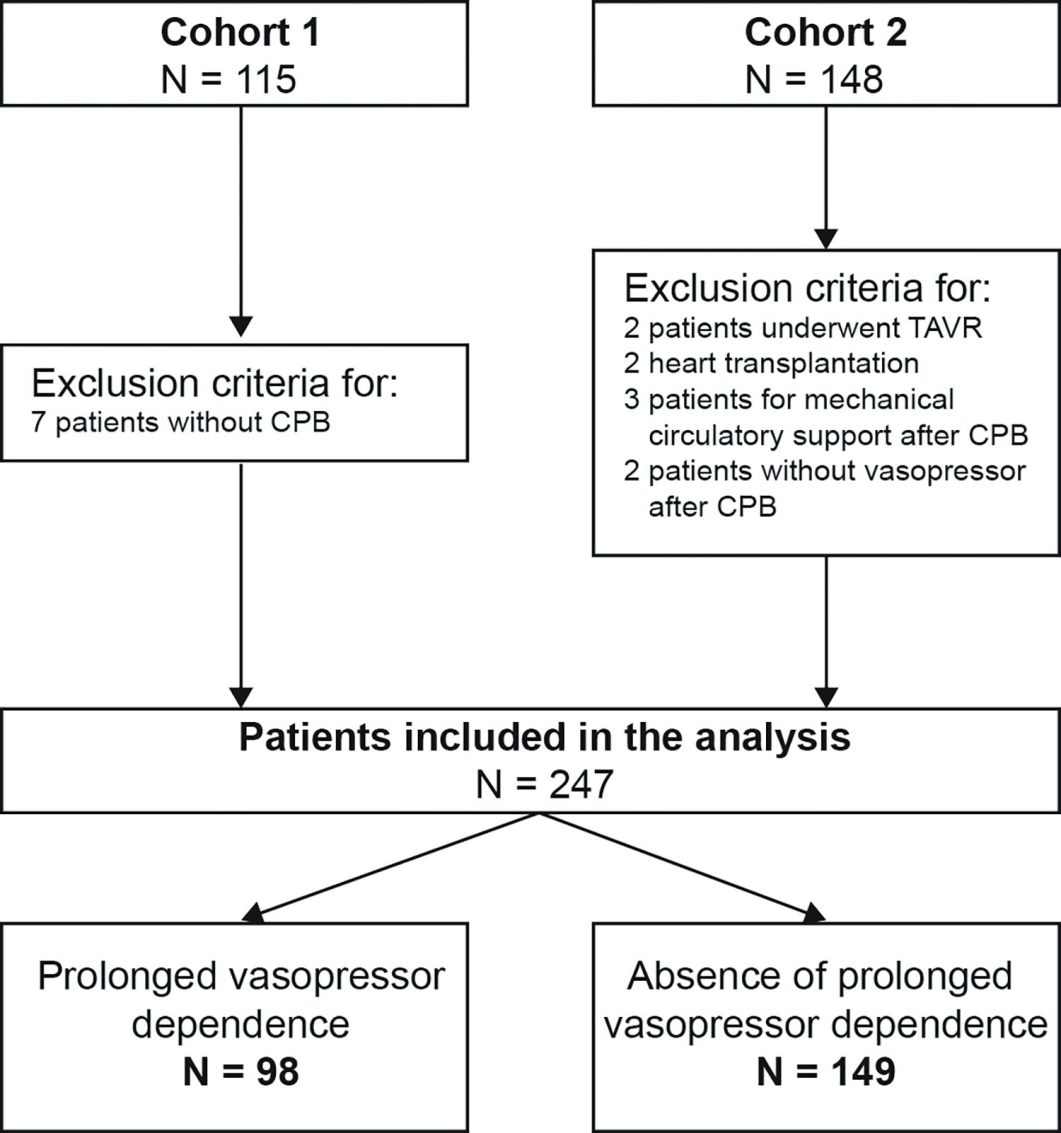
Flowchart of studied patients. Abbreviations: CPB, cardiopulmonary bypass; TAVR, transcatheter aortic valve replacement.

A total of 263 patients underwent cardiac surgery with CPB between November 2015 and July 2017. Sixteen patients were excluded. A total of 247 (93.9%) patients were included in the final analysis (Fig 1).

### Population characteristics and preoperative predictors

A total of 98 (39.7%) patients developed a prolonged cardiovascular pharmacological support (Table 1). These patients were older (67±12 *vs* 64±12 years, *P* < 0.02) and presented with a higher EuroSCORE II [3.1% (1.7–6.1) *vs* 1.8% (1.0–3.1), *P* < 0.0001], more frequent reduced LVEF of ≤ 30% [13 (13.3%) *vs* 1 (0.7%), *P* < 0.0001] and higher preoperative PH [severe 19 (22.6%) *vs* 7 (5.9%); and moderate 52 (61.9%) *vs* 60 (50.9%), *P* < 0.0001]. The preoperative use of loop diuretics [45 (45.9%) *vs* 33 (22.1%), *P* < 0.0001] as well as amiodarone [7 (7.1%) *vs* 1 (0.7%), *P* = 0.007] were more frequent in the prolonged prolonged cardiovascular pharmacological support group.

**Table 1:** Preoperative characteristics and intraoperative data of included patients.

	Absence of prolonged vasopressor dependence (n = 149)	Prolonged cardiovascular pharmacological support (n = 98)	*P*-value
**Characteristic**			
Male gender, n (%)	76 (51%)	45 (45.9%)	0.43
Age ± SD, years	64 ± 12	67 ± 12	0.02
EuroSCORE II, (%), median [IQR]	1.8 (1.0–3.1)	3.1 (1.7–6.1)	<0.0001
BMI, kg∕m^2^	29 ± 5	28 ± 5	0.08
**Comorbidities**			
Hypertension, n (%)	117 (78.5%)	76 (77.6%)	0.86
Diabetes, n (%)	48 (32.2%)	33 (33.7%)	0.81
Previous cardiothoracic surgery, n (%)	14 (9.4%)	16 (16.3%)	0.10
Previous myocardial infarction, n (%)	24 (16.1%)	19 (19.4%)	0.51
LV dilation, n (%)	19 (12.8%)	26 (27.4%)	0.004
LVEF ≤30%, n (%)	1 (0.7%)	13 (13.3%)	<0.0001
LV hypertrophy n (%)	39 (27.1%)	27 (29.4%)	0.71
Preoperative PH •No PH •Moderate PH (sPAP >30 but ≤55mmHg) •Severe PH (sPAP >55mmHg)	51 (43.2%) 60 (50.9%) 7 (5.9%)	13 (15.5%) 52 (61.9%) 19 (22.6%)	<0.0001
**Preoperative medications**			
ACE-ARB, n (%)	77 (51.7%)	56 (57.1%)	0.39
Beta-blockers, n (%)	83 (55.7%)	59 (60.2%)	0.48
CCB, n (%)	37 (25.0%)	22 (22.7%)	0.68
Loop diuretic n (%)	33 (22.2%)	45 (45.9%)	<0.0001
Amiodarone n (%)	1 (0.7%)	7 (7.1%)	0.007
**Intraoperative data**			
Mitral surgery, n (%)	25 (16.8%)	29 (29.6%)	0.02
Tricuspid surgery, n (%)	6 (4.0%)	14 (14.3%)	0.004
Multiple procedure surgery, n (%)	39 (26.2%)	41 (41.8%)	0.010
Difficult CPB weaning, n (%)	41 (27.5%)	51 (52.0%)	<0.0001
Duration of aortic clamp (min)	63 (41–81)	76 (49–101)	0.03
Duration of CPB time (min)	83 (65–108)	100 (75–129)	0.01
Duration of anesthesia (min), median [IQR]	290 (250–333)	308 (265–357)	0.03
Use of vasopressin, n (%)	42 (28.2%)	40 (40.8%)	0.04
Use of epinephrine, n (%)	21 (14.1%)	30 (30.6%)	0.002
Mean norepinephrine dose during the procedure (μg∕kg∕min)	0.04 ± 0.04	0.07 ± 0.05	<0.0001
Mean phenylephrine dose during the procedure (μg∕kg∕min)	0.10 ± 0.11	0.13 ± 0.13	0.04
Inhaled pulmonary vasodilators use before CPB, n (%)	23 (15.4%)	22 (22.5%)	0.16
Inhaled pulmonary vasodilators use after CPB, n (%)	35 (23.5%)	43 (43.9%)	0.0007
Packed red blood cells transfusion, n (%)	7 (4.7%)	9 (9.2%)	0.16
Intraoperative blood loss (mL), median [IQR]	325 (200–500)	362 (200–550)	0.36
Fluid intake during the procedure (mL)	2284 ± 879	2747 ± 1241	0.0007
Fluid intake during the procedure (mL/hr)	485 ± 226	525 ± 223	0.17
IFB during the procedure (mL)	1085 ± 894	1292 ± 1283	0.14
Use of loop diuretic during surgery, n (%)	16 (10.7%)	16 (16.3%)	0.20
Fluid removal using ultrafiltration during the procedure, n (%)	37 (24.8%)	36 (36.7%)	0.04
Upper lactate level after CPB (mmol/L)	1.6 ± 0.8	2.0 ± 1.2	0.005
Abnormal lactate level after CPB, n (%)	38 (25.5%)	37 (37.8%)	0.04
Minimal Hematocrit after CPB (%)	32.4 ± 4.0	30.5 ± 4.1	0.0005
pEEG monitoring use, n (%)	50 (33.6%)	34 (35.7%)	0.73
Hemodynamic monitoring by femoral-radial line, n (%)	109 (73.2%)	79 (80.6%)	0.18
Pulmonary artery catheter use, n (%)	113 (75.8%)	82 (83.7%)	0.14

Abbreviations: ACE, angiotensin converting enzymes; ARB, angiotensin II receptor blocker; BMI, body mass index; CCB, calcium channel blocker; CPB, cardiopulmonary bypass; EuroSCORE, European System for Cardiac Operative Risk Evaluation; ICU, intensive care unit; IFB, intraoperative fluid balance; IQR, interquartile range; LV, left ventricular; LVEF, left ventricular ejection fraction; PH, pulmonary hypertension; SD, standard deviation; sPAP, systolic pulmonary arterial pressure; pEEG, processed electroencephalography.

### Intraoperative predictors

Patients with prolonged cardiovascular pharmacological support more frequently had mitral surgery [29 (29.6%) *vs* 25 (16.8%), *P* = 0.0171], tricuspid surgery [14 (14.3%) *vs* 6 (4.0%), *P* = 0.004)], multiple procedures [41 (41.8%) *vs* 39 (26.2%), *P* = 0.01], more frequent difficult weaning from CPB [51 (52.0%) *vs* 41 (27.5%), *P* < 0.0001], a longer aortic cross clamp duration [76 (49–101) *vs* 63 (41–81) minutes, *P* = 0.0280], longer CPB duration [100 (75–129) *vs* 83 (65–108) minutes, *P* = 0.01] and a prolonged anesthesia duration [308 (265–357) *vs* 290 (250–333) minutes, *P* = 0.01] (Table 1). The use of vasopressin [40 (40.8%) *vs* 42 (28.2%), *P* = 0.04] and epinephrine [30 (30.6%) *vs* 21 (14.1%), *P* = 0.002] was also more common in those patients. Additionally, higher doses of norepinephrine (0.07±0.05 *vs* 0.04±0.04 μg/kg/min, *P* < 0.0001) and phenylephrine (0.13±0.13 *vs* 0.10±0.11 μg/kg/min, *P* = 0.04) were used during surgery in patients who subsequently developed prolonged cardiovascular pharmacological support. Patients with prolonged cardiovascular pharmacological support were more often exposed to inhaled pulmonary vasodilators (43 (43.9%) *vs* 35 (23.5%), *P* = 0.0007). Although patients with prolonged cardiovascular pharmacological support had a larger intraoperative fluid intake (2747±1241 *vs* 2284±879 mL, *P* = 0.0007), there was no significant difference when adjusted for the duration of the procedure (525±223 *vs* 485±226 mL/hr, *P* = 0.17) and in terms of fluid balance (1292±1283 *vs* 1085±894 mL, *P* = 0.14). In the vasopressor dependent groups, lactate levels after CPB were higher (2.0±1.2 *vs* 1.6±0.8 mmol/L, *P* = 0.005) and hematocrit levels after CPB were lower (30.5±4.1% *vs* 32.4±4.0%, *P* = 0.0005). A total of 175 patients (73.9%) had a pulmonary artery catheter; however, no association was noted between prolonged cardiovascular pharmacological support and the use of a pulmonary artery catheter [82 (83.7%) *vs* 113 (75.8%), *P* = 0.14], of continuous pEEG monitoring [34 (35.7%) *vs* 50 (33.6%), *P* = 0.73]. The use of a radial of femoral site for arterial pressure monitoring was similar between the groups [79 (80.6%) *vs* 109 (73.2%), *P* = 0.18].

### Postoperative predictors

In the postoperative period (Table 2), mechanical ventilation [5 hours (4–9) *vs* 4 hours (3–5), *P* < 0.0001], ICU [3 days (2–5) *vs* 1 day (1–2), *P* < 0.0001] and hospital length of stay [7 days (6–10) *vs* 5 days (4–7), *P* < 0.0001] were longer in patients with prolonged cardiovascular pharmacological support. A significant association was found between prolonged cardiovascular pharmacological support and the development of AKI [n = 43 (43.9%) *vs* n = 47 (31.5%), *P* = 0.049], AKI stage ≥2 [n = 13 (13.3%) *vs* n = 7 (4.7%), *P* = 0.0157], dialysis [n = 5 (6.4%) *vs* n = 1 (0.9%), *P* = 0.0324] and delirium [n = 21 (21.4%) *vs* n = 10 (6.7%), *P* = 0.001]. In addition, fluid management in the first 24 hours after surgery was marked by larger ICU fluid balance (937±1180 *vs* 135±766 mL, *P* < 0.0001) as well as a higher CFB (2229±1706 *vs* 1219±1110 mL, *P* < 0.0001).

**Table 2:** Clinical outcomes of included patients.

	Absence of prolonged vasopressor dependence (n = 149)	Prolonged cardiovascular pharmacological support (n = 98)	*P*-value
**Postoperative outcomes**			
Duration of vasopressor support after ICU admission (hrs), median [IQR]	3 (1–8)	48 (42–72)	<0.0001
Length of ICU stay (days), median [IQR]	1 (1–2)	3 (2–5)	<0.0001
Length of hospital stay (days), median [IQR]	5 (4–7)	7 (6–10)	<0.0001
Duration of ventilation (hrs), median [IQR]	4 (3–5)	5 (4–9)	<0.0001
AKI, n (%)	47 (31.5%)	43 (43.9%)	0.048
Severe AKI (Stage ≥ 2), n (%)	7 (4.7%)	13 (13.3%)	0.02
Dialysis	1(0.9%)	5 (6.4%)	0.03
Delirium (first 24 hours)	10 (6.7%)	21 (21.4%)	0.001
Fluid balance ICU day 1 (mL)	135 ± 766	937 ± 1180	<0.0001
CFB day 1 (mL)	1219 ± 1110	2229 ± 1706	<0.0001

Abbreviations: AKI, acute kidney injury based only on KDIGO criteria; CFB, cumulative fluid balance; ICU, intensive care unit; IQR, interquartile range; n, number.

### Independent risk factors for prolonged vasopressor dependence

**Fig 2:**
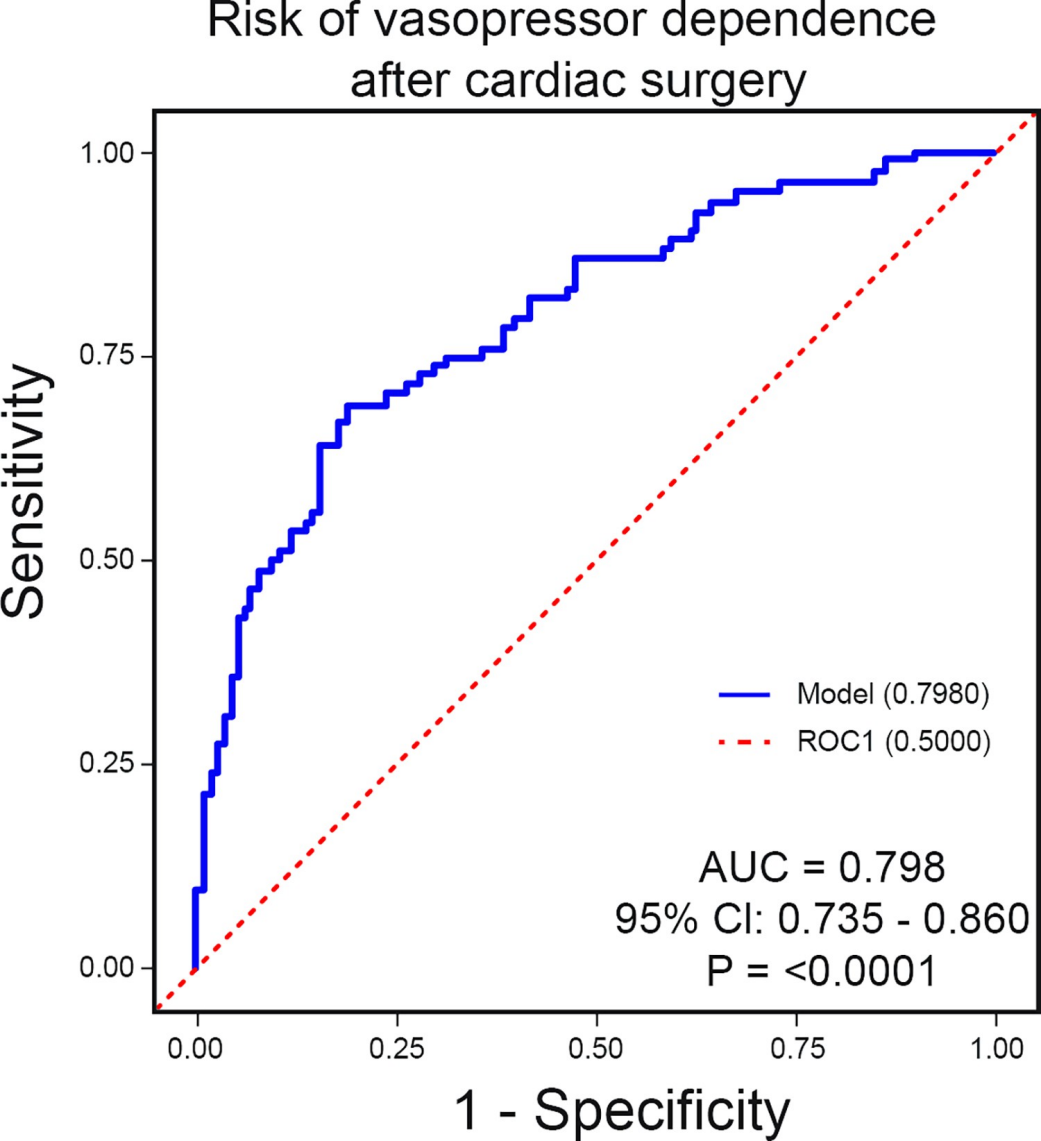
Receiver operating characteristic (ROC) curve representing the ability of the developed model to predict prolonged vasopressor dependence within the development cohort. Abbreviations: AUC, area under the curve; CI, confidence interval.

The univariate analysis between potential predictors and prolonged cardiovascular pharmacological support is detailed in S2 Table. The multivariate analysis is summarized in Table 3, which includes preexisting LVEF ≤30% (OR 9.52, 95% CI 1.14–79.25, *P* = 0.04), preoperative moderate and severe PH (moderate PH: OR 2.52, 95% CI 1.15–5.52, severe PH: OR 8.12, 95% CI 2.54–26.03, *P* = 0.002), and initial 24 hours CFB (OR 1.76, 95% CI 1.32–2.33 for 1 liter, *P* < 0.0001). The predictive model composed of the selected variables had adequately identified patients at risk of prolonged cardiovascular pharmacological support after cardiac surgery within the development cohort (area under the curve [AUC] 0.80, 95% CI: 0.74–0.86, *P* = <0.0001) (Fig 2). After internal bootstrap resampling, the internally validated C-statistic was estimated to be 0.79, 95 CI: 0.72–0.86 and the optimism in apparent performance was 0.07 with a 95% CI (0.07–0.08). The optimism-corrected area was therefore estimated at 0.72. Bootstrapping validation revealed a calibration slope of 0.87 (0.84; 0.90), indicating an overall agreement.

**Table 3:** Predictors of prolonged vasopressor dependence in multivariable logistic regression model.

Variable	Odds Ratio	(95% CI)	*P*-value
LVEF ≤ 30%	9.52	(1.14–79.25)	0.04
PH			0.002
Normal pulmonary pressure	Reference		
Moderate (sPAP >30 but ≤55mmHg)	2.52	(1.15–5.52)	
Severe (sPAP >55mmHg)	8.12	(2.54–26.03)	
CFB per 1L increase	1.76	(1.32–2.33)	<0.0001

Abbreviations: CFB, cumulative fluid balance; CI, confidence interval; LVEF, left ventricular ejection fraction; PH, pulmonary hypertension; sPAP, systolic pulmonary arterial pressure.

### Exploratory analysis

**Fig 3:**
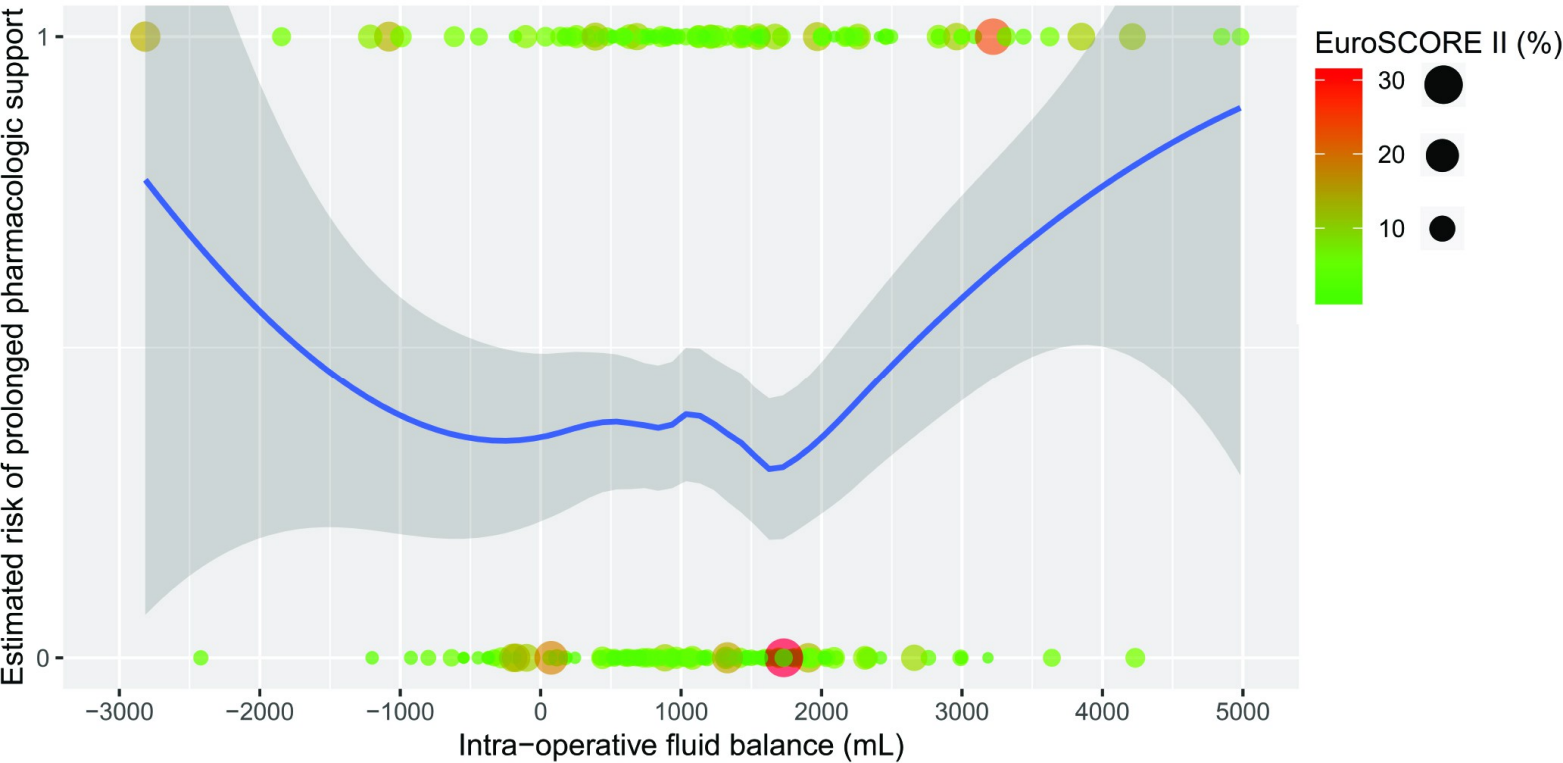
Estimation risk of postoperative vasopressor dependence in relation with intraoperative fluid balance and EuroSCORE II. Locally weighted scatterplot smoothing (LOESS) curve regression. LOESS fit line is shown with 95% of confidence intervals. The risk of postoperative prolonged vasopressor dependence appears to increase for both positive fluid balance and negative fluid balance during cardiac surgery.

Because the association between intraoperative fluid balance and the risk of prolonged cardiovascular pharmacological support did not satisfy the linearity of the logit assumption of logistic regression, the relationship was further explored. The risk of prolonged cardiovascular pharmacological support showed a U-shaped relationship indicating a tendency to increase for both positive and negative fluid balance during surgery, irrespective of EuroSCORE II (Fig 3) or CPB duration.

## Discussion

In this cohort of cardiac surgical patients, we found that preexisting severe LV systolic dysfunction, preoperative PH and postoperative fluid overload were independently associated with prolonged cardiovascular pharmacological support after cardiac surgery with CPB. The resulting model reliably identified patients who had prolonged cardiovascular pharmacological support within the studied sample. Similar to Weis *et al*. in their cohort of 1558 patients [11], we found that prolonged cardiovascular pharmacological support remains a frequent complication, with a prevalence of 40%, and is associated with adverse clinical outcomes as reported by others [3, 10, 24] such as AKI, prolonged mechanical ventilation, delirium and prolonged length of ICU and hospital stay.

Pre-operative PH and low LVEF are severity markers of the underlying severity of heart disease which may convey a higher risk of needing prolonged cardiovascular pharmacological support. As reported by Weiss [11] and Sun [2] reduced LVEF is an independent risk factor for prolonged vasoactive support. In addition, patients with preoperative LV dysfunction are known to have decreased myogenic reactivity to circulating catecholamines, thereby leading to a resistance to vasopressors [25]. PH is new and has not been previously reported as a risk factor associated with prolonged cardiovascular pharmacological support. However, PH and elevated pulmonary vascular resistance have been associated with a greater operative risk, a higher operative mortality and poor outcomes [26, 27]. PH can be present before or exacerbated after cardiac surgery [28]. Pulmonary reperfusion injury caused by CPB can exacerbate preexisting PH and lead to endothelial dysfunction, which in turn increases the pulmonary vascular resistance, elevated PAP and right ventricular (RV) dysfunction [29, 30]. Right heart failure is strongly associated with higher mortality after CPB [31]. Inhaled vasodilators could be used to reduce pulmonary resistance and improve RV dysfunction during CPB weaning [32–34]. Inhaled vasodilators used before and after CPB could represent a potential strategy in reducing vasoactive support. The hemodynamic efficacy combining inhaled prostacyclin and milrinone is close to 80% cardiac surgery [35, 36]. Reduction of vasoactive support has been by a retrospective analysis using this approach [37]. However, despite their hemodynamic advantages compared to intravenous agents [38], there is so far no evidence that they improve outcomes. In our study, inhaled vasodilators were used in 45 patients (18.2%) before CPB and in 78 patients (31.6%) after CPB. They were no difference in the rate of prolonged cardiovascular pharmacological support when inhaled vasodilators were administered before CPB; and while inhaled vasodilators were more commonly received after CPB in patient who developed prolonged cardiovascular pharmacological support, these were already most likely exhibiting a sub-optimal post-CPB course which prompted the treatment. On multivariate analysis, these inhaled vasodilators were found to be safe and not associated with prolonged cardiovascular pharmacological support.

A larger positive CFB 24 hours after CPB discontinuation was associated with a higher risk of prolonged cardiovascular pharmacological support. This is also new and no previous study has reported association between positive fluid balance and prolonged cardiovascular pharmacological support, but several studies have highlighted the association between fluid accumulation and postoperative complications [39–44]. In a randomized controlled trial of 573 cardiac surgical patients, Luciani *et al*. demonstrated that hemofiltration after CPB was associated with a lower prevalence of respiratory and gastrointestinal complications and transfusion requirements [45]. However, the authors did not report the intra- and postoperative fluid balance. Fluid overload may increase the risk of vasopressor dependence via multiple mechanisms. First, the shedding of endothelial glycocalyx in the context of CPB may be promoted by the release of brain natriuretic peptide from elevated cardiac filling pressure and lead to an alteration of vasomotor tone, causing vasodilation [46, 47]. In addition, fluid overload combined especially if combined with PH can lead to organ venous congestion and dysfunction, and bowel edema may lead to endotoxin translocation from the intestinal tract, exacerbating the general inflammatory response involved in the pathophysiology of vasoplegia [48, 49]. Supporting our findings, recently the appearance of portal pulsatility, a sign of venous congestion resulting from RV dysfunction, has been associated with fluid overload and prolonged vasoactive support after cardiac surgery in an international multicenter study [50]. Finally, acute normovolemic hemodilution anemia resulting in reduced hematocrit [9] is a risk factor for the vasoplegia. Such hematocrit reduction can be the result of a large fluid administration which decreases myocardial oxygen delivery and leads to the activation of vasodilatory pathways [51, 52]. When hemodynamic instability is related to RV dysfunction at the end of the CPB, fluid administration may worsen cardiac dysfunction by increasing myocardial wall tension and impairing LV filling by ventricular interdependence. Interestingly, we describe for the first time a U-shaped relationship between both negative and positive intraoperative fluid balance and an increase in the risk of prolonged vasopressor dependence after surgery, with an intraoperative optimal fluid balance between -500 mL and +1500 mL being associated to a lower risk. This relationship was observed even in some patients with a high preoperative risk using the EuroSCORE II or with a longer duration of CPB. This U-shaped relationship of the risk of atrial fibrillation in relation to fluid balance has also been observed in patients undergoing Cryo-Maze procedure [53]. Consistent with our observations, in a study of 18,084 critically ill patients, Balakumar *et al*. [54] reported that exposure to a positive or negative fluid balance compared to an even balance was associated with increased one-year mortality in critically ill patients.

Several limitations must be taken into consideration when interpreting our work. First, this is a single-center study with a small sample size, which limits the generalizability of the findings and our ability to detect additional potential risk factors that may be clinically significant. Furthermore, the observed mortality (1 death) was lower than predicted by the EuroSCORE II. Elective patients able to provide informed consent were likely to be included in this study which might explain this low mortality rate. We used a cut-off point of 24 hours to define prolonged cardiovascular pharmacological support which, while being relevant from a clinical standpoint, remains arbitrary. Furthermore, the duration of vasoactive agent may be influenced by MAP targets which may be individualized based on the clinical context. However, we observed that this cut-off identified patients more likely to develop postoperative complications which support its clinical relevance. Continuous cardiac output measurement was not available for all patients which precluded distinction between vasoplegia and LCOS. Despite the etiology, those vasoactive dependent patients were still required to stay in the ICU. In addition, the etiology of hemodynamic instability after cardiac surgery is often multifactorial, comprising LV and RV systolic and diastolic dysfunction and vasoplegia [55].

We identified 3 independent risk factors although it is unclear if these are amendable to intervention using inotropic agents, inhaled vasodilators, or optimized fluid management. Another important limitation is the potential for confounding by indication, particularly for fluid balance. Fluid administration is indicated in hypotensive, critically ill patients with signs of fluid responsiveness [56] without RV dysfunction. Therefore, the positive association between fluid balance and vasopressor dependence may only reflect medical intervention aimed at hemodynamic improvement after surgery. Finally, the predictive model produced requires validation in an external cohort.

## Conclusion

Prolonged cardiovascular pharmacological support after cardiac surgery remains a common problem associated with significant complications. Reduced LVEF, PH and a positive fluid balance were found to be independent risk factors in the context of cardiac surgery.

## Supporting information

S1 Table Definition of variables. (DOCX)

S2 Table Predictors of prolonged vasopressor dependence after cardiac surgery in univariable logistic regression analysis. (DOCX)

S1 Graphical abstract (PDF)

## Abbreviations

AKI: acute kidney injury
CFB: cumulative fluid balance
CPB: cardiopulmonary bypass
ICU: intensive care unit
IQR: interquartile range
LCOS: low cardiac output syndrome
LOESS: locally estimated scatterplot smoothing
LV: left ventricular
LVEF: left ventricular ejection fraction
PAP: pulmonary artery pressure
pEEG: processed electroencephalogram
PH: pulmonary hypertension
RV: right ventricular
SD: standard deviation
TEE: transesophageal echocardiography
VS: vasoplegic syndrome
