# Supplementary material for: Prolonged cardiovascular pharmacological support and fluid management after cardiac surgery
Source: PLoS One. 2023 May 11;18(5):e0285526. doi: 10.1371/journal.pone.0285526 (PMC10174538; doi:10.1371/journal.pone.0285526)
Supplement: S1 Table — (DOCX) [file pone.0285526.s001.docx]

**Supplementary Table S1**. Definition of Variables.

| **Variable** | **Definitions** |
| --- | --- |
| Body mass index (kg/m^2^) | Weight /Height^2^ |
| EuroSCORE II % [1] | A score based on EuroSCORE II calculator model |

| Comorbidities |  |
| --- | --- |

| Hypertension | History of treated or untreated hypertension |
| --- | --- |
| Diabetes mellitus | Diabetes with drug or insulin requirement |
| Previous cardiothoracic surgery | History of cardiothoracic surgery |
| Previous myocardial infarction | History of documented myocardial infarction |
| LV dilation | LVEDD >52mm documented by a preoperative echography |
| Reduced LVEF | LVEF ≤30%; LVEF was the last measured value reported before surgery by left ventriculography, echocardiography, or nuclear medicine; the lowest value was selected |
| LV hypertrophy | Defined as preoperative LV hypertrophy based on previous echocardiography and the American Society of Echocardiography criteria [2] |
| Preoperative PH [1] | Based on EuroSCORE criteria, PH is defined as a sPAP>30 mmHg or mPAP>25 mmHg measured by previous central venous catheterization. It is divided in two classes: Moderate PH: sPAP=31-55 mmHg and severe PH: sPAP>55mmHg  No PH: sPAP<30 mmHg [1] |
| Intraoperative variables |  |
| Pulmonary artery catheter use | The number of patients had pulmonary artery catheter used in OR |
| pEEG monitoring | Using either BIS or SedLine for continuous monitoring of brain function |
| Radial line for arterial pressure monitoring | Binary (yes/no) variable when blood pressure measured by radial or radial + femoral line [3] |
| Duration of anesthesia (min) | The time starting when the patient enters an OR until preparation surgical positioning can begin + the time starting when the dressing is finished and ending when the patient leaves the OR |
| Hemodynamic instability | Hemodynamic instability was defined as difficult separation from CPB; requires at least two different types of pharmacological agents (i.e., inotropes ± vasopressors ± inhaled agents) [4] |
| Vasopressors | Norepinephrine, vasopressin, dopamine and phenylephrine were defined as vasopressors |
| Inotropes | Milrinone, dobutamine and epinephrine were considered inotropic agents |
| Pulmonary vasodilatory agents | Inhaled prostacyclin, nitric oxide and milrinone alone or in combination |
| Complex surgery  Complex+ CABG = multiple procedure | Cardiac surgery involving repair or replacement of ≥ 2 valves without Valvular, aortic, or complex surgery associated with coronary revascularization |
| Difficult CBP weaning [5] | Required both vasopressors and inotropes or additional drugs like pulmonary vasodilatory agents or mechanical circulatory support such as the temporary re-institution of CPB. [4,6] |
| Intraoperative blood loss (ml) | Blood lost in suction bottles, weight of sponges CPB related and other sources |
| Minimal hematocrit after CPB | The lowest hematocrit value after the CBP and before ICU admission |
| Upper lactate level after CPB (mmol/L) | The upper lactate value after CPB and before ICU admission |
| Red blood cell transfusion | Binary (yes/no) variable, when red-blood-cell units were transfused in OR |
| IFB (ml) | Intraoperative fluid balance was calculated by subtracting urine output and blood loss (weight of the blood-soaked sponges during surgery and the amount of blood loss from fluid replacement (including crystalloids and colloids) and transfusion products; insensible losses from surgery and preoperative fasting were excluded from the calculation as previously described. [7] |
| Intraoperative fluid intake (ml) | The total of volume administrated during the procedure (including crystalloids, colloids and blood transfusion products |
| Abnormal hepatic venous flow Doppler [8] | Reversal S wave velocity or a ratio of S/D <1 |
| Portal pulsatility fraction [9]  Abnormal portal pulsatility fraction | The difference between systolic and diastolic velocities measured by abdominal echography  If the PF > 50%. = validated sign of portal hypertension [10] |
| Postoperative outcomes |  |
| Duration of vasopressor support after ICU admission | The time from the admission to the cessation of vasopressors |
| Death | Death within 30 days |
| Duration of ventilation (hours) | The number of hours between intubation upon arrival to the ICU and extubation |
| Length of hospital stay (days) | The duration of a single episode oh hospitalization and calculated by subtracting the day from the ICU admission from the day of discharge |
| AKI stage 1 and 2 [11] | According to KDIGO criteria the AKI stage 1 is defined as an elevation of serum creatinine >26μmol.L^-1^ in 48h or a reduction in urine output (˂0.5ml/kg/.h for 6-12h) and stage 2 defined as un elevation of serum creatinine 2.0-2.9 times baseline or a reduction in urine output (<0.5ml/kg/h for ≥ 12h). |
| ICU fluid balance day 1  CFB day 1 | Input – [urine output + blood loss] during the first 24h in ICU  Intraoperative fluid balance + ICU fluid balance day 1 |
| Delirium [7,12] | Defined as a diagnosis of acute confusion or encephalopathy at 24, 48- and 72-hours post-ICU arrival by the attending physician and/or a score of ≥4 on the Intensive Care Delirium Screening Checklist used by the nursing staff |

AKI, acute kidney injury; BIS, bispectral index; CABG, coronary artery bypass grafting; CFB, cumulative fluid balance; CPB, cardiopulmonary bypass; EuroSCORE, European System for Cardiac Operative Risk Evaluation; ICU, intensive care unit; IFB, intraoperative fluid balance; KDIGO, kidney disease improving global outcomes; LV, left ventricular; LVEDD, left ventricular end diastolic diameter; LVEF, left ventricular ejection fraction; mPAP, mean pulmonary arterial pressure; OR, operative room; pEEG, processed electroencephalography;

PF, portal pulsatility; PH, pulmonary hypertension; sPAP, systolic pulmonary arterial pressure.

**References**

**1.** Nashef SA, Roques F, Sharples LD, et al. EuroSCORE II. Eur J Cardiothorac Surg 2012;41:734-744.

**2.** Lang RM, Badano LP, Mor-Avi V, et al. Recommendations for cardiac chamber quantification by echocardiography in adults: an update from the American Society of Echocardiography and the European Association of Cardiovascular Imaging. J Am Soc Echocardiogr 2015;28:1-39.e14.

**3.** Fuda G, Denault A, Deschamps A, et al. Risk Factors Involved in Central-to-Radial Arterial Pressure Gradient During Cardiac Surgery. Anesth Analg 2016;122:624-632.

**4.** Denault AY, Bussieres JS, Arellano R, et al. A multicentre randomized-controlled trial of inhaled milrinone in high-risk cardiac surgical patients. Can J Anesth 2016;63:1140-1153.

**5.** Denault AY, Tardif JC, Mazer CD, Lambert J; BART Investigators. Difficult and complex separation from cardiopulmonary bypass in high-risk cardiac surgical patients: a multicenter study. J Cardiothorac Vasc Anesth 2012;26:608-616.

**6.** Denault AY. Difficult separation from cardiopulmonary bypass: importance, mechanism and prevention. Université‚ de Montréal; 2010.

**7.** Mailhot T, Cossette S, Lambert J, et al. Delirium After Cardiac Surgery and Cumulative Fluid Balance: A Case-Control Cohort Study. J Cardiothorac Vasc Anesth 2019;33:93-101.

**8.** Hulin J, Aslanian P, Desjardins G, et al. The Critical Importance of Hepatic Venous Blood Flow Doppler Assessment for Patients in Shock. A A Case Reports 2016;6:114-120.

**9.** Beaubien-Souligny W, Eljaiek R, Fortier A, et al. The Association Between Pulsatile Portal Flow and Acute Kidney Injury after Cardiac Surgery: A Retrospective Cohort Study. J Cardiothorac Vasc Anesth 2018;32:1780-1787.

**10.** Westra SJ, Zaninovic AC, Vargas J, et al. The value of portal vein pulsatility on duplex sonograms as a sign of portal hypertension in children with liver disease. AJR. Am J Roentgenol 1995;165:167-172.

**11.** Burton JO, Goldsmith DJ, Ruddock N, et al. Renal association commentary on the KDIGO (2017) clinical practice guideline update for the diagnosis, evaluation, prevention, and treatment of CKD-MBD. BMC Nephrol 2018;19:240.

**12.** Tse L, Schwarz SK, Bowering JB,et al. Incidence of and Risk Factors for Delirium After Cardiac Surgery at a Quaternary Care Center: A Retrospective Cohort Study. J Cardiothorac Vasc Anesth 2015;29:1472-1479.
