# Supplementary material for: Prolonged cardiovascular pharmacological support and fluid management after cardiac surgery
Source: PLoS One. 2023 May 11;18(5):e0285526. doi: 10.1371/journal.pone.0285526 (PMC10174538; doi:10.1371/journal.pone.0285526)
Supplement: S2 Table — (DOCX) [file pone.0285526.s002.docx]

**Supplementary Table S2.** Predictors of Prolonged Vasopressor Dependence after Cardiac Surgery in Univariable Logistic Regression Analysis.

| **Variables** | **Univariable** | | |
| --- | --- | --- | --- |
| **Preoperative** | Odds Ratio | (95%CI) | *P*-value |
| Age | 1.03 | (1.00-1.05) | 0.02 |
| Male gender | 0.82 | (0.49-1.36) | 0.43 |
| BMI | 0.96 | (0.91-1.01) | 0.08 |
| EuroSCORE II > 5% | 3.59 | (1.86-6.95) | 0.0001 |
| LVEF ≤ 30% | 22.63 | (2.91-176.04) | 0.003 |
| LV dilation | 2.56 | (1.32-4.95) | 0.005 |
| Previous myocardial infarction | 1.25 | (0.65-2.44) | 0.51 |
| Hypertension | 0.95 | (0.51-1.75) | 0.86 |
| Previous cardiothoracic surgery | 1.88 | (0.87-4.06) | 0.11 |
| Severe PH  Moderate vs No  Severe vs No | 3.40  10.64 | (1.67-6.94)  (3.69-30.70) | <0.0001 |
| Use of ACE or ARB before surgery | 1.25 | (0.75-2.08) | 0.40 |
| Use of Beta-blockers before surgery | 1.20 | (0.72-2.02) | 0.48 |
| Use of CCB before surgery | 0.88 | (0.48-1.61) | 0.68 |
| Use of amiodarone before surgery | 11.38 | (1.38-93.93) | 0.02 |
| Use of loop diuretics before surgery | 2.98 | (1.71-5.20) | 0.0001 |
| Use of IV heparin before surgery | 1.37 | (0.78-2.39) | 0.27 |
| **Intraoperative** |  |  |  |
| Anesthesia duration, per hour increase | 1.39 | (1.11-1.75) | 0.004 |
| Duration of CPB, per hour of increase | 1.44 | (1.03-2.01) | 0.03 |
| Use of continuous limited pEEG monitoring | 1.10 | (0.64-1.88) | 0.73 |
| Use of radial line for arterial pressure monitoring | 1.53 | (0.82-2.83) | 0.18 |
| Mean norepinephrine dose during the procedure  > 0.1 µg/kg/min vs < 0.1 µg/kg/min | 3.99 | (1.73-9.18) | 0.0011 |
| Phenylephrine during the procedure (total μg/min) | 1.01 | (0.99-1.03) | 0.17 |
| Inhaled anesthesia agents during the procedure (total ml) | 1.00 | (0.99-1.01) | 0.97 |
| Use of propofol, (per mg increase) | 0.999 | (0.998-1.000) | 0.02 |
| Ultrafiltration | 1.76 | (1.01-3.06) | 0.046 |
| Use of desmopressin during surgery | 2.35 | (1.10-5.06) | 0.03 |
| Blood loss during surgery, (per 100 ml increase) | 1.09 | (0.995-1.190) | 0.06 |
| Aortic valve surgery | 0.80 | (0.47-1.34) | 0.39 |
| Mitral valve surgery | 2.09 | (1.13-3.84) | 0.02 |
| Pulmonary valve surgery | 0.59 | (0.18-1.94) | 0.39 |
| Tricuspid surgery | 3.97 | (1.47-10-73) | 0.007 |
| Aortic thoracic surgery | 1.06 | (0.43-2.58) | 0.90 |
| Combined surgery | 2.03 | (1.18-3.49) | 0.01 |
| Difficult weaning from CPB | 2.86 | (1.67-4.88) | 0.0001 |
| **Before CPB** |  |  |  |
| Abnormal hepatic venous Doppler flow | 1.08 | (0.52-2.25) | 0.84 |
| PPF  > 50% vs ≤ 30%  30% to 50% vs ≤ 30% | 2.21  2.21 | (0.58-8.48)  (1.08-4.53) | 0.06 |
| Use of pulmonary vasodilators | 1.59 | (0.83-3.04) | 0.16 |
| **After CPB separation to end of surgery** |  |  |  |
| Use of pulmonary vasodilators | 2.55 | (1.47-4.41) | 0.0009 |
| Abnormal lactate level ≧ 2 mmol/L (Yes vs No) | 1.47 | (0.84-2.58) | 0.18 |
| Hematocrit decrease > 20% from baseline | 1.56 | (0.92-2.64) | 0.10 |
| **First 24 hours in ICU** |  |  |  |
| Abnormal hepatic venous Doppler flow | 1.63 | (0.94-2.83) | 0.08 |
| PPF  > 50% vs ≤ 30%  30% to 50% vs ≤ 30% | 1.29  1.65 | (0.58-2.91)  (0.85-3.20) | 0.31 |
| Abnormal lactate level ≧ 2 mmol/L (Yes vs No) | 1.73 | (1.03-2.89) | 0.038 |
| Cumulative fluid balance in the first 24h (per L) | 1.78 | (1.41-2.24) | <0.0001 |

ACE, angiotensin converting enzyme; ARB, angiotensin II receptor blocker; BMI, body mass index;

CCB, calcium channel blocker; CPB, cardiopulmonary bypass; EuroSCORE, European System for Cardiac Operative Risk Evaluation; ICU, intensive care unit; IV, intra-venous; LV, left ventricular; LVEF, left ventricular ejection fraction; pEEG, processed electroencephalography; PH, pulmonary hypertension;

PPF, portal pulsatility fraction.
