# Supplementary material for: Prolonged cardiovascular pharmacological support and fluid management after cardiac surgery
Source: PLoS One. 2023 May 11;18(5):e0285526. doi: 10.1371/journal.pone.0285526 (PMC10174538; doi:10.1371/journal.pone.0285526)
Supplement: S1 Graphical abstract — (PDF) [file pone.0285526.s003.pdf]

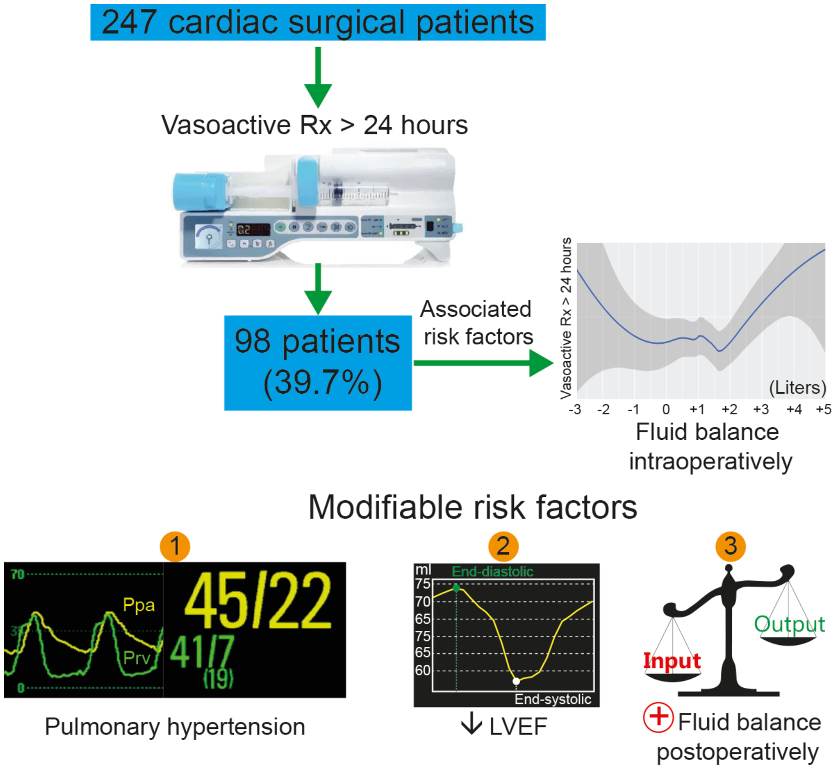

247 cardiac surgical patients
Vasoactive Rx > 24 hours
98 patients (39.7%)
Associated risk factors
Vasoactive Rx > 24 hours
(Liters)
-3 -2 -1 0 +1 +2 +3 +4 +5
Fluid balance intraoperatively
Modifiable risk factors
1
70
Ppa
Prv
0
45/22
41/7
(19)
Pulmonary hypertension
2
ml
End-diastolic
75
70
65
75
65
60
End-systolic
↓ LVEF
3
Input
Output
Fluid balance postoperatively
